# Profibrotic Biomarkers Correlate with Clinical Presentation and Outcome in Cardiac Transthyretin Amyloidosis

**DOI:** 10.3390/ijms262110714

**Published:** 2025-11-04

**Authors:** Selina J. Hein, Fabian aus dem Siepen, Arnt V. Kristen, Stefan Schönland, Ute Hegenbart, Katrin Rein, Hugo A. Katus, Norbert Frey, Jennifer Furkel, Mathias H. Konstandin, Maximilian Knoll

**Affiliations:** 1 Department of Cardiology, Angiology and Pneumology, University Hospital Heidelberg, 69120 Heidelberg, Germany; 2 DZHK (German Center for Cardiovascular Research), Site Heidelberg/Mannheim, 69120 Heidelberg, Germany; 3 Department of Hematology, Oncology and Rheumatology, Heidelberg University, 69120 Heidelberg, Germany; 4 Department of Radiation Oncology, German Cancer Research Center (DKFZ) and Heidelberg University Hospital (UKHD), Heidelberg Ion-Beam Therapy Center (HIT), 69120 Heidelberg, Germany; 5 German Cancer Consortium (DKTK) Core-Center, German Cancer Research Center (DKFZ), 69120 Heidelberg, Germany; 6 Division of Molecular & Translational Radiation Oncology, Heidelberg Faculty of Medicine (MFHD) and Heidelberg University Hospital (UKHD), Heidelberg Ion-Beam Therapy Center (HIT), 69120 Heidelberg, Germany; 7 Heidelberg Institute of Radiation Oncology (HIRO), National Center for Radiation Research in Oncology (NCRO), German Cancer Research Center (DKFZ) and Heidelberg University Hospital (UKHD), 69120 Heidelberg, Germany; 8 CCU Translational Radiation Oncology, National Center for Tumor Diseases (NCT), German Cancer Research Center (DKFZ) and Heidelberg University Hospital (UKHD), 69120 Heidelberg, Germany

**Keywords:** cardiac transthyretin amyloidosis, fibrosis, outcome, risk prediction, remodeling

## Abstract

In transthyretin cardiac amyloidosis (ATTR-CA), misfolded transthyretin accumulates in the myocardium, leading to wall thickening and interstitial fibrosis. Recently published in vitro studies revealed direct effects of transthyretin on the structure, function, and gene expression of cardiac fibroblasts. Therefore, we hypothesized that biomarkers known to modulate myocardial remodeling might be clinically valuable in ATTR-CA and may improve risk stratification in ATTR-CA. To analyze this hypothesis, we evaluated 14 fibrosis-related biomarkers (EN-RAGE, IGFBP-1, -2, -3, -4, -6, FGF-23, MMP-2, -7, -9, -13, TIMP-2, -4, and RAGE-AGE) in 125 patients using Luminex multiplex assays. The study cohort consists of 14 asymptomatic gene carriers (ATTRv-asymp), 47 symptomatic hereditary (ATTRv-CA), 43 wild-type Transthyretin amyloidosis (ATTRwt) patients, and 21 were healthy controls (ctrl). Associations of fibrotic biomarkers and clinical routine data with clinical outcomes—cardiac decompensation (DMP) and transplantation/death (HTX)—were assessed via hierarchical cluster analysis, regression, and prediction modeling. We found that ATTR-CA patients showed distinct biomarker profiles compared to controls. Several markers (e.g., MMP-7, RAGE-AGE, IGFBP-1, FGF-23, TIMP-2) were significantly associated with both endpoints. Cluster analysis identified a high-risk phenotype (Cluster 2) with worse renal function, greater myocardial thickening, and elevated NT-proBNP, hsTNT. Prediction modeling revealed IGFPB-1, -3, -4 and -6 as well as FGF-23, TIMP-2, and RAGE/AGE as the best predictive parameters for cluster assignment. Taken together, these findings confirm our hypothesis that fibrosis-related biomarkers are associated with adverse outcomes in ATTR-CM. Profibrotic mediators such as IGFBP-1, FGF-23, and TIMP-2 may, therefore, provide additional prognostic information beyond established cardiac biomarkers and may reflect underlying fibrotic remodeling pathways.

## 1. Introduction

Heart failure (HF) remains one of the leading causes of morbidity and mortality in developed countries and is characterized by heterogeneous underlying mechanisms. While patients with reduced ejection fraction (HFrEF) typically present with dilated ventricles and reduced systolic function due to the loss of cardiomyocytes [1], the pathomechanisms leading to heart failure with preserved ejection fraction (HFpEF) are more complex [2]. Common findings in HFpEF patients include low-grade inflammatory changes, cardiomyocyte hypertrophy, and cardiac fibrosis [3,4]. In their review article, Mesquita et al. demonstrate the heterogeneity of the pathomechanisms leading to HFpEF, emphasizing the need for further detailed information about distinct HFpEF patient subgroups [5]. Among the diverse etiologies of HFpEF, cardiac amyloidosis represents a distinct and increasingly recognized subgroup, particularly in elderly individuals. With the growing availability of diagnostic tools and disease-modifying therapies, early identification of ATTR cardiomyopathy (ATTR-CM) has become important. Therefore, the development of novel sensitive diagnostic tools and screening strategies leading to early recognition of the disease and timely initiation of treatment became crucial [6].

Transthyretin cardiac amyloidosis (ATTR-CA), caused either by hereditary transthyretin mutations (ATTRv) [7] or wild-type transthyretin depositions (ATTRwt) [8], is an infiltrative cardiomyopathy marked by progressive diastolic dysfunction, wall thickening, and restrictive filling patterns. Typical morphological changes observed in echocardiography lead to the diagnosis of ATTR-CA. Myocardial hypertrophy, detected by elevated posterior wall (PW) and intraventricular septum (IVS), diastolic dysfunction, indicated by elevated E/e’ parameters in echocardiography, and reduced longitudinal function, indicated by decreased MAPSE and shortened longitudinal strain, are usually depicted. The severity and prognosis of ATTR cardiomyopathy are characterized by the cardiac biomarkers N-terminal prohormone of brain natriuretic peptide (NT-proBNP), high-sensitivity troponin T (hs-TnT), and glomerular filtration rate (GFR). Depending on the levels of these biomarkers, patients are classified into clinical risk classes according to Gillmore and Grogan’s staging system [8,9].

Although amyloid burden is central to the disease pathogenesis of ATTR-CA, accumulating evidence indicates that interstitial fibrosis and extracellular matrix remodeling contribute significantly to cardiac dysfunction and possibly to prognosis [10]. This was recently emphasized by experimental studies showing that transthyretin fibrils directly alter fibroblast function in vitro, suggesting that circulating fibrosis-related mediators may reflect active disease pathways [11].

We, therefore, hypothesized that circulating profibrotic biomarkers are elevated in ATTR-CA and are associated with disease severity and outcome. To test this, we analyzed a broad panel of fibrosis-related proteins in a well-characterized ATTR-CA cohort and applied clustering and prediction modeling to identify biomarker signatures linked to clinical risk.

### Biomarker in Cardiac Remodeling

Based on the hypothesis that extracellular fibrillary amyloid deposits may disrupt extracellular protein homeostasis, the expressions of matrix metalloproteinases (MMPs) and their tissue inhibitors (TIMPs) were analyzed in an ATTR-PN mouse model. In this model, RNA interference (RNAi)-induced downregulation of mutant TTR led to decreased MMP-2 and MMP-9 expression [12]. This finding was supported by the demonstration that MMP-2 and MMP-9 are upregulated in patients with ATTR-PN [12,13]. In ATTR-CA, elevated MMP levels are known in AL amyloidosis, but little is known about MMPs in ATTR-CA so far [14]. Therefore, this study investigated different MMP and TIMP levels in ATTR-CA.

Another potential mechanism of amyloid toxicity is the receptor for advanced glycation endproduct (RAGE)-dependent pathway. RAGE is a multi-ligand receptor known to regulate chronic inflammatory responses. It exists in membrane-bound and soluble isoforms. Fibrillary amyloid, advanced glycation endproducts (AGE), and EN-RAGE/S100A12 are among its high-affinity ligands [15]. Moreover, RAGE has been shown to increase in nerve and gastrointestinal tissue obtained from patients with familial ATTR polyneuropathy [16,17]. In this study, we analyzed the soluble form of the RAGE receptor and its ligands AGE (RAGE/AGE) and EN-RAGE/S100A12 in patients’ blood plasma.

Furthermore, the role of insulin-like growth factor (IGF) pathways was investigated in a mouse model of Alzheimer’s disease (AD), another illness caused by amyloid deposition. In this model, increased levels of TTR were associated with the absence of Alzheimer’s disease [18]. Additionally, several insulin-like growth factor (IGF) pathways, including insulin-like growth factor binding proteins (IGFBPs), have been described in heart failure, hypertrophic cardiomyopathy, and hypoxic cardiomyocytes [19,20,21,22,23]. Therefore, we hypothesized that a similar pathway might be activated in ATTR-CA and decided to evaluate the levels of IGFBP-1, 2, 3, 4, and 6.

Fibroblast growth factor 23 (FGF-23) is a phosphaturic hormone elevated in patients with renal insufficiency and associated with cardiac hypertrophy and pathological cardiac remodeling [24,25]. Furthermore, FGF-23 has been shown to predict outcomes in amyloidosis and acute heart failure [26,27]. For this reason, we included FGF-23 as a potential biomarker in this study.

**Figure 1:**
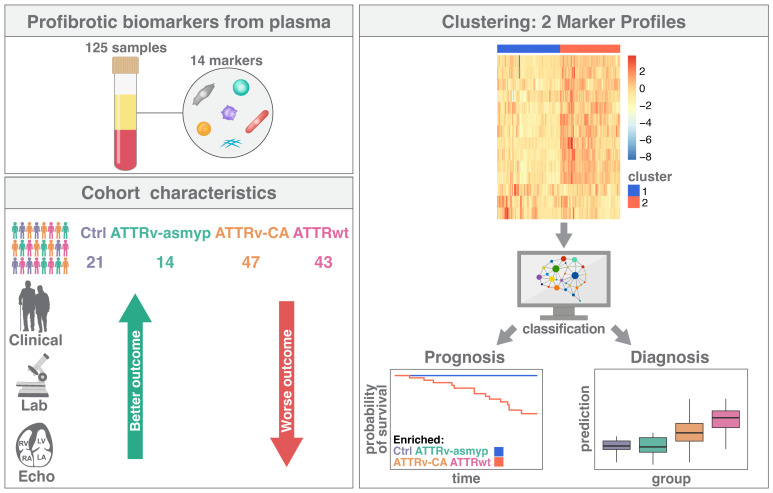
Graphical presentation of the study design. Ctrl: controls, ATTRv-asymp: asymptomatic gene carriers of a hereditary mutation in the transthyretin gene, ATTRv-CA: patients with hereditary ATTR cardiomyopathy, ATTRwt: patients with wild-type cardiac ATTR amyloidosis.

As the aforementioned studies indicate the multifactorial influences of transthyretin-induced myocardial remodeling, we decided to analyze a broad panel of biomarkers involved in fibrotic pathways. We use cluster analyses derived from data mining methods to identify patients at high cardiovascular risk and to gain insight into pathophysiological pathways and risk prediction in ATTR patients. The study design is demonstrated graphically in Figure 1.

## 2. Results

### 2.1. Study Population

A total of 125 individuals including 21 healthy controls participated in this study. None of the patients included in the study had acute infections, chronic inflammatory states (e.g., autoimmune diseases), or were taking immunosuppressive medication. The asymptomatic gene carrier group (ATTRasym) consisted of 14 patients. Diagnosis was ascertained by family history and subsequent genotyping for ATTR mutations. These patients did not present any clinical or subclinical signs or symptoms of amyloidosis in echocardiography, echocardiogram or laboratory testing. The remaining 90 study participants had hereditary or wild-type amyloidosis. In 49 patients presenting with manifest cardiac amyloidosis, the diagnosis was confirmed invasively via myocardial biopsy. In the remaining 51 patients, the diagnosis was confirmed non-invasively via specific myocardial storage in 99m-TC-DPD bone scintigraphy, along with concomitant serological exclusion of AL amyloidosis. According to the examination results, patients were grouped into the following categories: ATTRwt (*n* = 43), symptomatic ATTRv-cardiomyopathy (CM) (*n* = 47), and asymptomatic ATTRv-CM (*n* = 14).

The ATTRv-CM group consists of patients with the following mutations: Val30Met (*n* = 16), Val20Ile (*n* = 11), Ile107Val (*n* = 5), Leu58His (*n* = 5), Cys10Arg (*n* = 2), Val122Ile (*n* = 3), Ala25Thr (*n* = 1), Ile84Asn (*n* = 1), Ile107Phe (*n* = 1), Arg34Gly (*n* = 1), and Thr106Arg (*n* = 1). The following mutations were identified in the asymptomatic mutation carriers: Val30Met (*n* = 5), Val20Ile (*n* = 5), Val122Ile (*n* = 1), Ile87Val (*n* = 1), Cys10Arg (*n* = 1), and Ile84Thr (*n* = 1). Table 1 presents the clinical characteristics of the study participants.

ATTRwt patients were significantly older and predominantly male. Furthermore, the ATTRwt group was more likely to be treated with diuretics, beta blockers, and ACE inhibitors. Atrial fibrillation was more prevalent in ATTRwt patients than in the other patient groups, and ATTRwt patients had a higher prevalence of bundle branch blocks. Additionally, diabetes was more frequently recorded in their medical history as a comorbid disease. Serologically, ATTRwt patients had significantly higher hsTnT and NT-proBNP levels, as well as a lower glomerular filtration rate (GFR), compared to the other two groups. These results led to higher clinical risk classes according to the Gillmore Staging system [9]. Echocardiography revealed that ATTRwt and ATTRv patients had more severe diastolic dysfunction than patients in the other two groups.

### 2.2. Profibrotic Biomarkers Are Elevated in Patients with Symptomatic ATTR Amyloidosis (ATTRv-CA)

**Figure 2:**
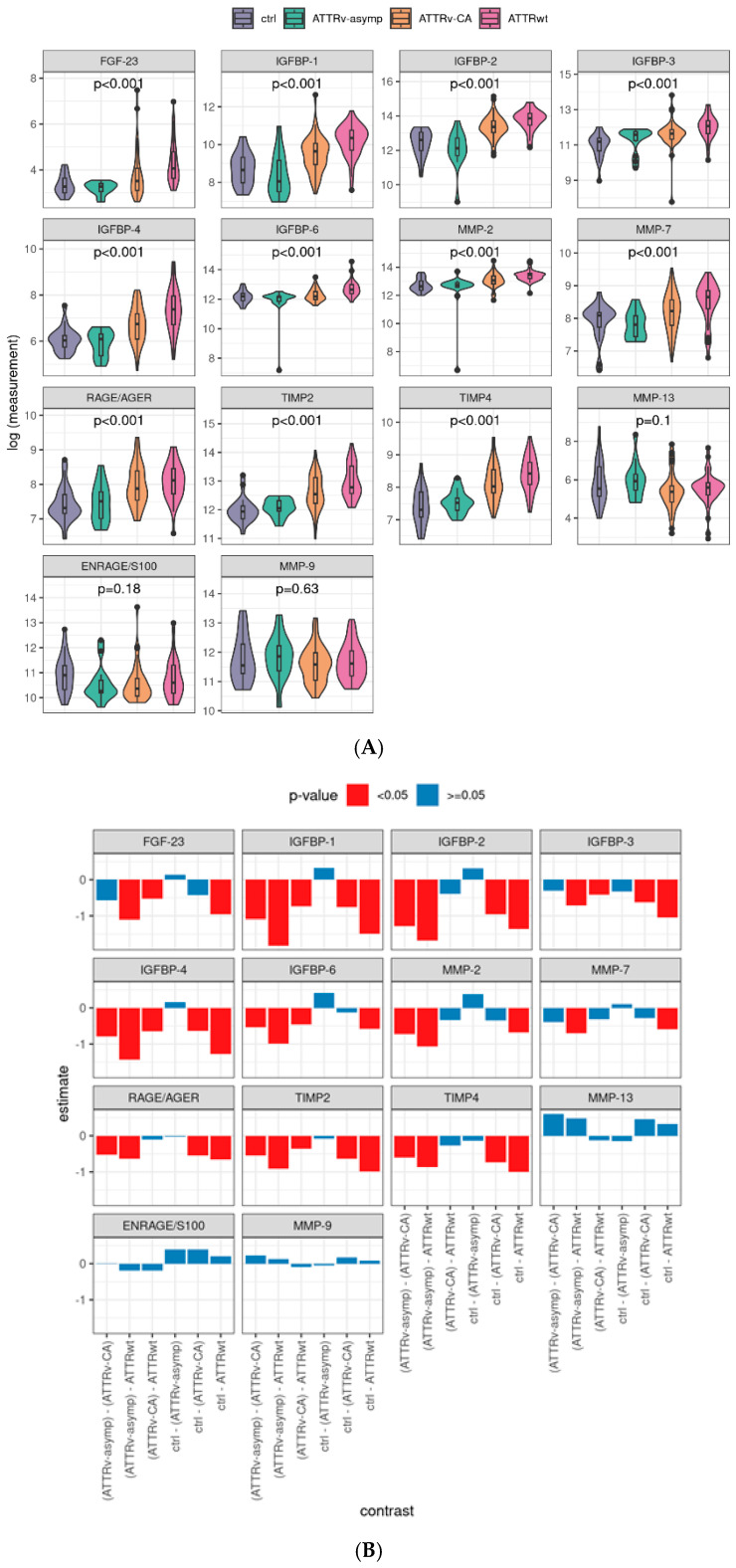
Distribution of serum profibrotic markers by study group and corresponding contrasts: Logarithmized values of the plasma levels of analyzed profibrotic biomarkers and the linear model *p*-values (**A**). Post hoc contrasts and its *p*-values in bar charts, pairwise tests with Tukey *p*-value adjustment. (**B**) ENRAGE: glycation endproducts binding protein, FGF: fibroblast growth factor; IGFBP: insulin-like growth factor binding protein; MMP: matrix metalloproteinase; RAGE: receptor for advanced glycation endproducts; AGE: advanced glycation endproducts; TIMP: tissue inhibitor of metalloproteinases. Ctrl: controls, ATTRv-asymp: asymptomatic gene carriers of a hereditary mutation in the transthyretin gene, ATTRv-CA: patients with hereditary ATTR cardiomyopathy, ATTRwt: patients with wild-type cardiac ATTR amyloidosis.

RAGE/AGE, FGF-23, IGFBP-1, IGFBP-2, IGFBP-3, IGFBP-4, IGFBP-6, MMP-2, MMP-7, TIMP-2, and TIMP-4 were significantly different between the ctrl and amyloidosis subgroups (*p* < 0.001, Figure 2A); ENRAGE/S100A12, MMP-13, and MMP-9 showed no significant difference. Post hoc tests confirmed that symptomatic ATTR patients (ATTRv-CA and ATTRwt) showed mostly significantly increased levels of profibrotic markers (Figure 2B), whereas asymptomatic individuals (ATTRv-asym) vs. controls (ctrl) showed no difference. Interestingly, a natural hierarchy could be observed corresponding to the expected level of symptoms, e.g., for IGFBP-1: ctrl/ATTRv–asymp < ATTRv-CA < ATTRwt levels. Furthermore, it is remarkable that FGF-23 presented significant differences between both control groups and ATTRwt as well as between ATTRwt and ATTRv-CA but not between control groups and ATTRv-CA, indicating that this biomarker might be valuable for specific detection of ATTRwt.

### 2.3. Multiple Profibrotic Biomarkers Are Associated with Cardiovascular Events in ATTR-CA

Next, the profibrotic parameters were tested for their ability to predict cardiovascular events using Cox-PH models. Follow-up after the index event was available for 121 of 125 patients (96.8%). The overall median follow-up period was 17.0 (95% CI: 15.0–19.0) months, 9.0 (95% CI: 3.8–14.2) for death/heart transplantation (HTX), and 29.0 (95% CI: 21.7–36.3) months for decompensation. During the follow-up period, 18 patients died or were listed for urgent heart transplantation. Furthermore, 12 patients were hospitalized due to cardiac decompensation and required parenteral diuretic administration for recompensation.

**Figure 3:**
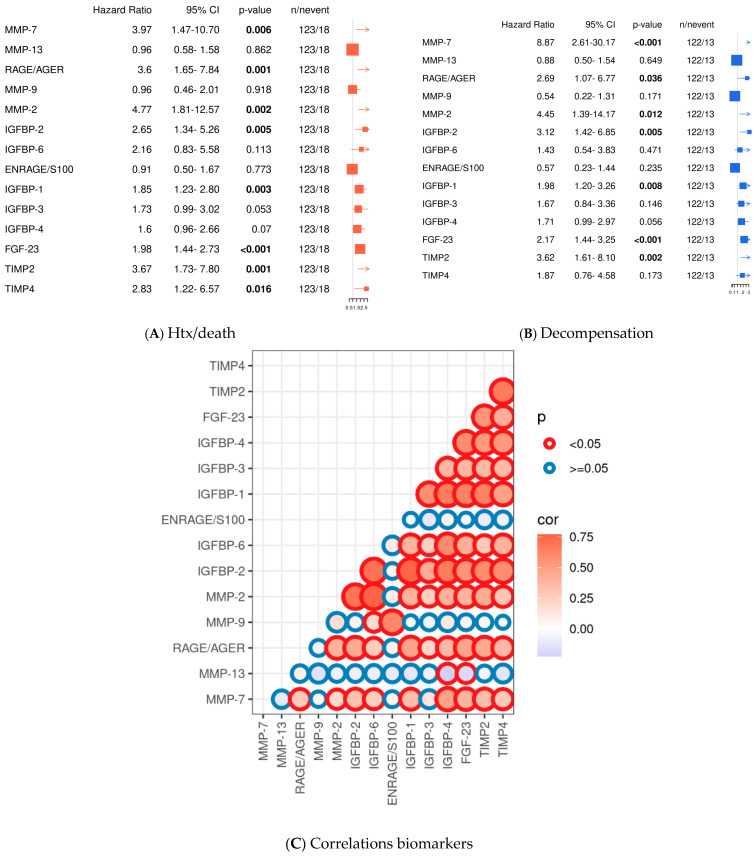
Univariate analysis of profibrotic biomarkers and Pearson correlations. Cox-PH models for continuous variables for the endpoint death/transplantation (HTX) (**A**); univariate analysis for the endpoint decompensation (**B**); Pearson correlation analyses of the tested biomarkers presented graphically with its *p*-values and correlation coefficients. Circle lines in red demonstrate significant differences between the biomarkers, blue circle lines depict non-significant differences (*p* ≥ 0.05). Circle fillings represent correlation coefficients: blue depicts low correlation coefficients and red higher correlation coefficients. The size of the circle reflects the level of significance. (**C**). Significant values are indicated by parameters printed in bold letters, with a level of significance of *p* < 0.05.

The majority of parameters elevated in ATTR patients compared to controls were also positively associated with the cardiovascular endpoint of death/transplantation (MMP-7: *p* = 0.006, RAGE/AGE: *p* = 0.001, MMP-2: *p* = 0.002, FGF-23: *p* < 0.001, and IGFBP-1: *p* = 0.003; IGFBP-2: *p* = 0.005; IGFBP-3: *p* = 0.053; TIMP-2: *p* = 0.001; and TIMP-4: *p* = 0.016). These parameters were also associated with decompensation (MMP-7: *p* < 0.001; RAGE/AGE: *p* = 0.036; MMP-2: *p* = 0.012; FGF-23: *p* < 0.001; IGFBP-1: *p* = 0.008; IGFBP-2: *p* = 0.005; and TIMP-2: *p* = 0.002). In contrast, IGFBP-3 (*p* = 0.146) and TIMP-4 (*p* = 0.173) showed only a trend for the endpoint decompensation (see Figure 3A). Univariate analyses of clinical data showed that, as expected, clinical signs of advanced ATTR-CA (elevated NYHA class, IVS, PW, TNT, and NT-proBNP, as well as reduced GFR) were significantly associated with both endpoints (HTX/death and decompensation), whereas MAPSE was only associated with cardiac decompensation. Interestingly, elevated C-reactive protein (CRP) and leukocytosis, indicating a proinflammatory state, were also associated with both cardiovascular endpoints (see Supplementary Figure S2).

### 2.4. Cluster Analyses Reveal Patients at High Cardiovascular Risk

**Figure 4:**
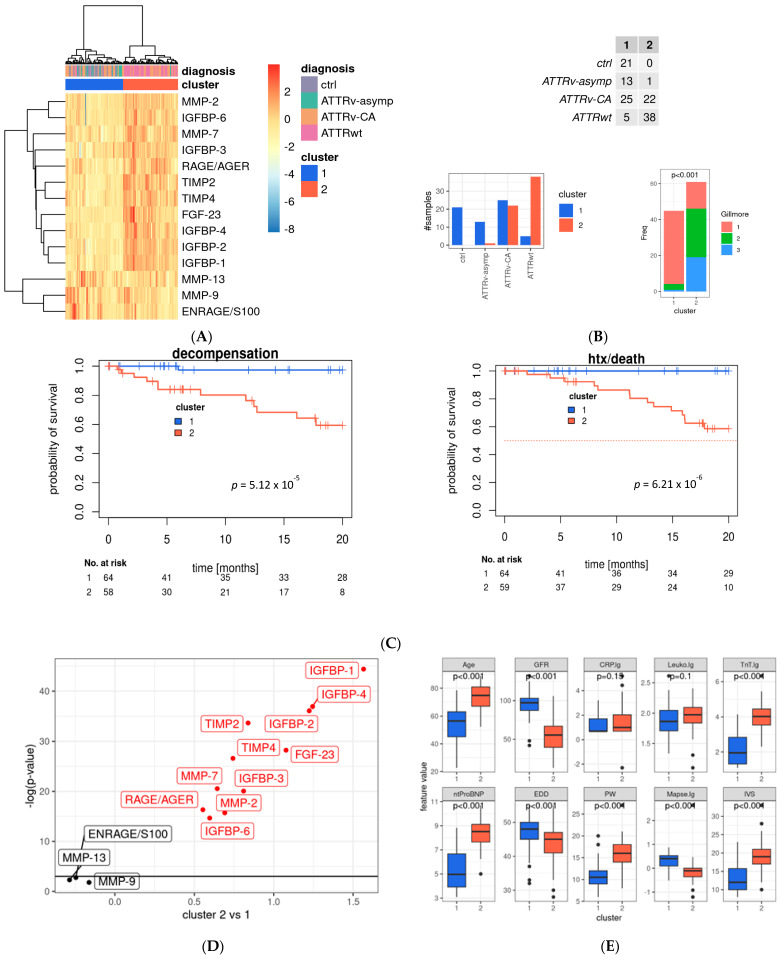

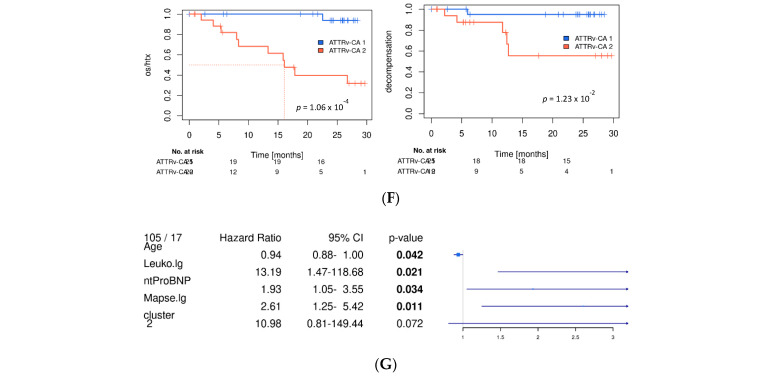
Main profibrotic marker profiles in the evaluated cohort. (**A**) Heat map with hierarchical clustering of analyzed profibrotic biomarkers; rows represent the tested biomarker, and columns represent the patient sample; hierarchical clustering was performed using Ward’s D2 distance. The dendrograms illustrate the clustering structure. Color gradients indicate relative values, with red representing higher and blue representing lower expression levels. Identified clusters are marked in red (Cluster 1) and blue (Cluster 2) and were used for further analysis. Data is batch-normalized, log- and z-transformed. (**B**) Distribution of study group and two main clusters and distribution by Gillmore Stage (chi-quadrat Test). (**C**) Kaplan–Meier curves of the whole study cohort for the endpoint death or heart transplantation. (**D**) Volcano plot for association of profibrotic markers and Cluster 1 and 2 (linear model analysis). (**E**) Distribution of echocardiographic and laboratory parameters as well as patient characteristics between main cluster groups. (**F**) Kaplan–Meier survival curves for the ATTRv-CA subgroup for the endpoint death/htx (**left**) and decompensation (**right**), blue: Cluster 1, red: Cluster 2. Significant values are indicated by parameters printed in bold letters, with a level of significance of *p* < 0.05. (**G**) Multivariate analysis for variables retained after model selection (see Supplementary Figure S3); MMP: matrix metalloproteinase; ENRAGE: glycation endproducts binding protein; IGFBP: insulin-like growth factor binding protein; FGF: fibroblast growth factor; TIMP: tissue inhibitor of metalloproteinases; RAGE: receptor for advanced glycation endproducts; AGER: advanced glycation endproducts receptor.

Since the tested fibrotic markers are part of various cardiovascular pathways and undergo regulatory changes and counteraction, we decided to investigate our dataset further using data mining methods. Only complete datasets were included, data was log-transformed, and ComBat batch correction was performed to minimize technical variability (see Supplementary Figure S1). We performed hierarchical cluster analysis (Figure 4A): all healthy controls and most asymptomatic gene carriers were assigned to Cluster 1 (which showed favorable prognostic outcome; see below), whereas most patients suffering from ATTRwt were allocated to Cluster 2. Patients with ATTRv-CA were partially assigned to Cluster 1 or 2 (see Figure 4B).

The most widely used and established clinical risk classification for patients with ATTR-CA is the Gillmore Staging system, which is determined by patients’ GFR and NTpro-BNP levels. Patients with low mortality risk are assigned to Gillmore Stage 1, whereas patients with higher mortality risk are assigned to Gillmore Stage 2 and 3 [9]. Our cluster analysis assigned most patients with Gillmore Stage 1 to the favorable Cluster 1. Patients allocated to Cluster 2, however, presented with approximately one-third of Gilmore Stage 1, 2, and 3 indicating that our cluster analysis might identify further patients at risk who belong to Gillmore Stage 1 or 2. In detail, in the ATTRv-CA group, 25 patients were assigned to low-risk Cluster 1, and 22 were assigned to high-risk Cluster 2. In the ATTRwt group, 5 patients were assigned to Cluster 1 and 38 to Cluster 2. Finally, 13 asymptomatic gene carriers who belong to the ATTRv-asymp were assigned to Cluster 1 and only one was assigned to Cluster 2 according to the profibrotic biomarker levels (see Figure 4B).

Thus, our cluster analysis might detect patients with cardiac involvement and distinguish those at high cardiovascular risk from those at lower risk. The prognostic value of the cluster was demonstrated using Kaplan–Meier curves (see Figure 4C,F as well as Supplementary Figure S3C for subgroup analyses), which show separation of low- and high-risk patients by the two clusters, as well as for the endpoints of death or transplantation and decompensation.

Analyses of differences in profibrotic biomarkers in Cluster 1 and 2 revealed that most of the tested biomarkers differ significantly between the two clusters. IGFBP1-4 and -2 as well as FGF-23 were significantly elevated in Cluster 2 in comparison to Cluster 1. In contrast, TIMP-2, -4, MMP-7, -2, IGFBP3, -6 as well as RAGE/AGE were lower in Cluster 2 compared to Cluster 1. Only ENRAGE/S100, MMP9 and MMP-13 did not differ significantly between the two clusters (see Figure 4D).

Analyses of established risk factors for cardiac amyloidosis in Cluster 1 and 2 revealed that individuals assigned to the unfavorable Cluster 2 were significantly older and had significantly higher levels of high-sensitivity troponin T (hsTNT) and N-terminal pro-brain natriuretic peptide (NT-proBNP), as well as pronounced myocardial hypertrophy, as indicated by elevated posterior wall (PW) and intraventricular septum (IVS) thickness. Consecutively, enddiastolic diameter (EDD), mitral annular plane systolic excursion (MAPSE), and GFR were reduced (see Figure 4E). As patients with ATTRv-CA were assigned half to Cluster 1 and 2, we confirmed the survival prediction force of our clusters in this subgroup and found stable highly significant differences in the Kaplan–Meier curves of this subgroup (see Figure 4F).

#### Analyses of Tafamidis Subgroup

To further explore the potential influence of disease-modifying therapy, the ATTRv subgroup was stratified according to the tafamidis treatment status. During the study period Tafamidis was only approved for the treatment of ATTR polyneuropathy in hereditary amyloidosis. Therefore, the patients received 20 mg Tafamidis daily. As shown in Supplementary Figure S5, patients receiving tafamidis showed a trend toward improved outcomes for the combined endpoint and the endpoint death/htx (*p* = 0.19). While the effect did not reach statistical significance, Kaplan–Meier analyses demonstrated a favorable separation of survival curves for tafamidis-treated patients. The best separation of Kaplan–Meier curves was attained for the endpoint of cardiac decompensation (*p* = 0.008). Baseline characteristics including age, hsTNT, NYHA class, and Karnofsky performance score (KPS) revealed that patients receiving Tafamidis were older but did not differ in KPS, NYHA class, and hsTNT levels (see Supplementary Figure S5). These findings support the concept that tafamidis may attenuate disease progression even in the early stages of hereditary ATTR amyloidosis, aligning with previously reported clinical benefits in larger cohorts.

### 2.5. Stepwise Selection of Multivariate Analyses Revealed Age, Leucocyte Count, NT-proBNP and Cluster Assignment as Independent Risk Factors

To further investigate if the identified clustering parameter (fibrosis signature) adds prognostic information to single marker information alone, we assessed correlations between markers per cluster (Supplementary Figure S3A) and we performed stepwise model selection using all markers and the cluster assignment (Supplementary Figure S3B).

Correlation analysis showed that FGF-23 was positively correlated with most parameters tested. Namely, this was confirmed for IGFBP-1,2,3,4 and 6, as well as MMP-2, -9 and RAGE/AGE. Moreover, IGFBP-1 was positively correlated with IGFBP-2,3,6, RAGE/AGE, MMP-2, and TIMP-2. RAGE/AGE was positively correlated with IGFPB-2 and TIMP-2 and negatively with IGFBP-3.

In the model selection analysis, for death/transplantation endpoint, RAGE/AGER, IGFBP-1, FGF-23, and the cluster assignment parameters were retained; for the decompensation endpoint, MMP-7, -9, -13, IGFBP-1, -2, -4, TIMP2, and the cluster assignment were selected (see Supplementary Figure S3B). Thus, the combined evaluation of profibrotic biomarkers (reflected in the cluster assignments) adds prognostic information to single-parameter assessments.

Similar results occurred when evaluating cluster assignment in the context of patient characteristics (age, NYHA class), laboratory parameters (CRP, Leukocytosis, GFR, hsTNT, NTpro-BNP), and echocardiographic parameters (MAPSE, EDD, PW, IVS) (see Figure 4G)—the parameter clustering for the death/transplantation endpoint were still retained (Figure 4G). The final model contains age, leukocytosis, NT-proBNP level, and clustering (HR: 10.98, *p*-value = 0.072).

Taken together, this result supports the dominant prognostic role of classical markers but also suggests that composite profibrotic biomarkers may carry additional biological information which might be analyzed in larger cohorts and/or subgroups of ATTR-CA in further studies and might be valuable for pathophysiological studies.

### 2.6. Supervised Prediction Modeling Identified IGFBP-1, -3, -4, -6 as Well as FGF-23, TIMP-2, MMP2 and AGE/RAGE as Best Markers to Predict Cluster Assignment

**Figure 5:**
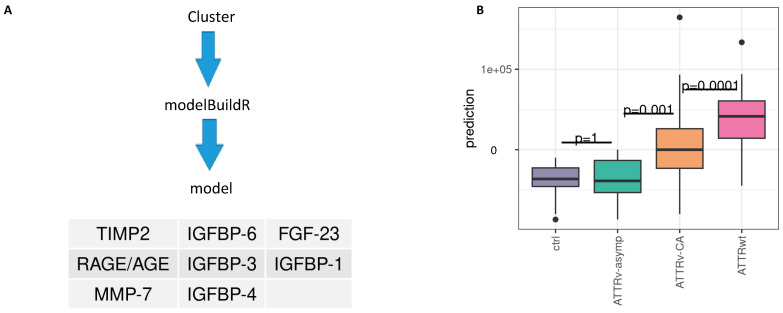
Prediction of cluster assignment. (**A**) Model building and retained features. (**B**) Predicted model values and differences between subtypes, Tukey *p*-value adjustment; IGFBP: insulin-like growth factor binding protein; FGF: fibroblast growth factor; TIMP: tissue inhibitor of metalloproteinases. RAGE: receptor for advanced glycation endproducts; AGE: advanced glycation endproducts.

Our unsupervised cluster analysis based on 14 circulating profibrotic biomarkers revealed two distinct patient subgroups assigned to Cluster 1 and 2. To simplify clinical usability of our findings we aimed to identify the most relevant predictors of cluster affiliation. We subsequently applied the R package modelBuildR (v3.6.3) for robust feature selection (logistic regression: cluster 1 vs. 2). This supervised modeling approach reduced the biomarker set and identified IGFBP-1, -3, -4, -6 as well as FGF-23, TIMP-2, MMP2, and AGE/RAGE as the key variables responsible for cluster assignment. Using these eight biomarkers, predicted log odds show significant differences between ctrl/ATTRv-asym, ATTRv-CA and ATTRwt groups (Figure 5B), indicating that they also represent strong discriminators between the identified subgroups (see Figure 5A and Supplementary Figure S4). Thereby differentiation between ATTRv-CA and ATTRwt, as well as ATTRv-asymp and ATTRv-CA might be feasible with this reduced set of markers.

## 3. Discussion

In our prospective monocentric study, we analyzed the plasma levels of 14 fibrosis-associated biomarkers in 125 patients with hereditary (ATTRv) or wild-type transthyretin (ATTRwt) amyloidosis and demonstrated their significant association with clinically measured plasma levels and cardiovascular outcome in ATTR amyloidosis (ATTR-CA). Our findings suggest that profibrotic signaling pathways are not only altered in ATTR-CA patients but also play a crucial role in disease progression in addition to established risk prediction parameters.

Several biomarkers—including IGFBP-1, IGFBP-2, IGFBP-3, FGF-23, MMP-2, MMP-7, TIMP-2, and RAGE/AGE—were significantly elevated in patients with cardiac involvement and independently associated with either heart failure decompensation or death/transplantation. Particularly, IGFBP-1 and FGF-23 emerged as important parameters indicating elevated risk of death/htx and IGFBP-4 for cardiac decompensation in stepwise model selection, underscoring their potential as novel markers of myocardial remodeling and outcome in ATTR-CA.

### 3.1. Role of Fibrosis in the Pathophysiology of ATTR-CA

Our results are in line with recent histopathological and functional studies highlighting the fibrotic component of ATTR-CA. A very recent pivotal study by Milburn et al. showed that extracellular matrix expansion in ATTR-CA, rather than amyloid burden alone, impairs contractile force and diastolic function, pointing to fibrosis as a determinant of mechanical dysfunction [28]. Furthermore, in vitro studies by Dittloff et al. revealed that both tetrameric and fibrillar TTR disrupt cytoskeletal architecture, adhesion, and transcriptional profiles of human cardiac fibroblasts, ultimately promoting a profibrotic and proinflammatory phenotype [29]. Together, these findings support the concept that TTR deposits contribute to cardiac fibrosis not merely by passive amyloid deposits but through activation of cellular signaling.

Among the profibrotic pathways implicated, the insulin-like growth factor axis has emerged as a key modulator of cardiac stress and fibrosis. IGFBP-1 and IGFBP-2 have previously been shown to predict mortality in heart failure [19,20,22] and are elevated in other fibrotic diseases, such as idiopathic pulmonary fibrosis [30,31]. Their upregulation in our ATTR-CA cohort supports their role as systemic mediators of myocardial remodeling. Particularly, IGFBP-1 emerged as potent parameter in stepwise model selection as well as in prediction modeling for cluster assignment.

The phosphaturic hormone FGF-23 is another promising biomarker linked to left ventricular hypertrophy, myocardial fibrosis, and cardiovascular mortality, especially in renal dysfunction [32,33,34]. Our finding that FGF-23 levels independently predict cardiac decompensation supports its potential as a mechanistic link between amyloid cardiomyopathy and maladaptive myocardial remodeling.

Matrix metalloproteinases (MMPs) and their tissue inhibitors (TIMPs) are central regulators of extracellular matrix turnover. Prior studies showed distinct MMP/TIMP patterns in AL- versus ATTR-CA, with MMP-9 being more prominent in AL-CA [14,35]. In our cohort, MMP-2 and MMP-7 were significantly elevated in ATTR-CA and associated with adverse outcomes, while MMP-9 was not. This differential expression pattern supports the hypothesis of organ- and amyloid-type-specific matrix regulation.

RAGE/AGE signaling, previously associated with amyloid toxicity in familial polyneuropathy, cystic fibrosis, and Alzheimer’s disease [15,16,17,36], was also significantly altered in ATTR-CA. Our findings confirm prior in vitro data showing that RAGE can bind TTR fibrils and trigger NF-κB-mediated inflammation, potentially linking amyloid deposition to fibrotic and inflammatory pathways [37].

### 3.2. FGF-23 and Its Cardiorenal Interaction

FGF-23 is known to be an independent risk factor in numerous cardiovascular diseases, including coronary heart disease, heart failure and hypertrophic cardiomyopathy. Although FGF-23 plasma concentrations are influenced by renal function, our findings suggest that its association with outcome in ATTR cardiomyopathy extends beyond kidney function alone, similar to other cardiovascular diseases [38]. In our cohort, FGF-23 correlated not only with glomerular filtration rate (GFR) but also with cardiac biomarkers such as NT-proBNP and hs-TnT (see Supplementary Figure S3), reflecting the cardiorenal interaction in amyloid cardiomyopathy. The high-risk cluster identified in our analysis combined impaired renal function with advanced myocardial remodeling and elevated cardiac biomarkers, suggesting that FGF-23 captures the integrated burden of systemic disease and not only the renal impairment. In our multivariate analyses, the predictive value of association to cluster 2 remained significant even after integrating FGF-23 to the multivariate models (see Supplementary Figure S6B).

### 3.3. Age- and Stage-Dependence of Biomarker Performance

In our cohort, ATTRwt patients were older and presented with more advanced cardiac involvement and renal dysfunction, whereas ATTRv patients more often underwent earlier diagnosis (commonly via family screening) and therapy initiation. The differences in age and disease stage increase the range of circulating profibrotic biomarkers and weaken their prognostic discrimination in younger/earlier-stage ATTRv. While our unsupervised clusters did stratify outcomes within the ATTRv subgroup, effect sizes were smaller and multivariate model stability was limited by fewer events. However, the prognostic value of Cluster 2 remained significant even when age and sex was added as covariates to the multivariate analyses (see Supplementary Figure S6A).

Pathophysiologically, the literature suggests that myocardial extracellular expansion in amyloid cardiomyopathy is not simply a linear function of amyloid load alone: for example, fibrosis and matrix remodeling appear to contribute increasingly in advanced disease [10], and modeling data indicate that functional decline may accelerate with higher burden of deposition and structural damage [39]. Hence, at early stages (typical in younger ATTRv cohorts), biomarker gradients may be modest, while in later stages (more common in older ATTRwt), the impact of profibrotic activity and matrix expansion may amplify the prognostic signal, potentially indicated by QRS progression in ECG [40]. These observations underscore the need for age- and stage-specific cut-offs. Future work may also consider modeling non-linear trajectories of deposition, fibrosis, biomarkers, and outcome risk.

### 3.4. Novelty of This Study

The novelty of our study lies in the comprehensive multimarker evaluation of fibrosis-associated pathways in a well-characterized ATTR cohort, integrating unsupervised and supervised analytical approaches to identify prognostically relevant biomarker signatures. While individual profibrotic markers such as FGF-23 or IGFBPs have been previously studied in heart failure, their combined assessment in ATTR-CA and the identification of specific cluster-based phenotypes have not, to our knowledge, been reported yet. Our findings extend current knowledge by demonstrating that circulating fibrosis-related mediators not only reflect disease severity but also stratify clinical risk beyond conventional staging systems, suggesting distinct molecular remodeling patterns within the spectrum of ATTR cardiomyopathy.

### 3.5. Limitations

This study is limited by the moderate number of events during follow-up, which constrained the number of variables that could be included in multivariate models. As most of the patients included in this study were diagnosed non-invasively by serological exclusion of monoclonal gammopathy and typical myocardial enhancement in skeletal scintigraphy, ethically we were not able to assess myocardial tissue samples for fibrosis or amyloid burden and thereby were not able to correlate our data with histological findings. Moreover, as TTR-lowering therapies for ATTR cardiomyopathy were still under clinical evaluation at the time of recruitment, the influence of therapy on biomarker trajectories would need to be addressed in subsequent studies.

Although in vivo and in vitro studies suggest that inflammatory and fibrosis-inducing pathways play a role in the pathophysiology of amyloidosis [11,28], the potential confounding effects of age and comorbidities in the study cohort should not be neglected. Due to the natural course of the disease, the ATTRwt group was older than the ATTRv group, but aging itself goes along with increased oxidative stress and a chronic, low-grade proinflammatory state, both of which may alter circulating profibrotic biomarker levels [41]. Moreover, chronic conditions common in elderly patients—such as diabetes mellitus, coronary heart disease, renal dysfunction, or hypertension—may further activate inflammatory and fibrotic pathways, thereby contributing to the observed biomarker alterations. Although we adjusted for age in multivariate analyses, residual confounding factors cannot be fully excluded. Future studies with age- and comorbidity-matched controls are warranted to delineate the independent contribution of amyloid-related fibrosis from age-associated remodeling processes.

### 3.6. Conclusions and Future Directions

Our study highlights the importance of fibrosis-related biomarkers in the pathophysiology and prognosis of cardiac ATTR amyloidosis. Future studies should focus on validating these findings in larger, multicenter cohorts and explore the utility of these biomarkers in monitoring disease progression and therapeutic response—particularly in the context of specific anti-amyloid and antifibrotic therapies.

## 4. Materials and Methods

### 4.1. Study Population

From August 2016 to October 2019, all patients present at our tertiary referral center for amyloidosis at Heidelberg University Hospital were screened and evaluated for participation in this study. All patients were willing to donate blood for the study and signed their informed consent approved by the Heidelberg Ethics Committee (S-485-2016), in accordance with the Declaration of Helsinki. One individual did not want to participate in the study. One hundred twenty-nine patients fulfilled the inclusion criteria. The inclusion criteria include an age range of 18 to 90 years, having a diagnosis of ATTRv-CA, being an asymptomatic gene carrier of a pathognomonic ATTRv or having a diagnosis of ATTRwt. Patients whose diagnosis remained unclear (*n* = 4) were not eligible to participate in the study. Furthermore, patients who received TTR-lowering therapy or participated in an interventional clinical trial were not eligible. Thus, a total of 125 patients could be included in the study. Additionally, 21 healthy volunteers with normal biomarker (NT-proBNP, hsTNT, and CRP) and echocardiographic results were asked to donate blood for the study. Blood samples were obtained via routine venipuncture during a medical visit at our tertiary referral center for amyloidosis. One additional lithium heparin monovette (4.9 mL, Sarstedt) was drawn and centrifuged. The plasma was then aliquoted and stored at −80 °C until the Luminex^®^ Multiplex Assays (R&D Systems, Minneapolis, MN, USA) were performed. Luminex data were then analyzed using a Luminex^®^ MAGPIX fluorescent imager.

### 4.2. Echocardiography

Echocardiography was performed using M-mode, Doppler, two-dimensional (2D) imaging, and global longitudinal strain (GLS) analyses. The ejection fraction was calculated using 2D echocardiography imaging, and diastolic dysfunction was graded according to the current guidelines of the American Society of Echocardiography [42]. Grades II and higher were considered significant diastolic dysfunction.

### 4.3. Follow-Up

Endpoint follow-up was performed by a phone call 12 months after the patient was included in the study. The following pre-specified endpoints were evaluated: death or cardiac transplantation (HTX/death), and cardiac decompensation (cDCMP).

### 4.4. Luminex Assay

The plasma concentrations of the 14 fibrosis-associated parameters evaluated in this study were analyzed using the Luminex MAGPIX system (R&D Systems, Minneapolis, MN, USA). The manufacturer designed four different multibeads. These multibeads carry analyte-specific antibodies. They were used for the multiplex immunoassays. Measurements were conducted according to the manufacturer’s protocols and recommendations. Based on the expected plasma concentrations of the biomarkers and the available bead configurations, four assays were necessary to test all biomarkers. TIMP-2 and TIMP-4 were analyzed using the Human TIMP Luminex^®^ Performance Assay (R&D Systems, Minneapolis, MN, USA). In this assay, plasma samples were analyzed at a dilution of 1:50. The remaining assays used Human Magnetic Luminex^®^ Assays (catalog numbers LXSAHM-05 and LXSAHM-03). IGFBP-1, IGFBP-3, IGFBP-4, and FGF-23 were analyzed using human plasma at a 1:2 dilution with the provided kit buffer (catalog number LXSAHM-05). EN-RAGE, IGFBP-6, MMP-2, MMP-9, and IGFBP-2 were analyzed using a 1:100 dilution of plasma samples (catalog number LXSAHM-05), while MMP-7, RAGE/AGE, and MMP-13 were analyzed without further plasma sample dilution (catalog number LXSAHM-03). Biomarker concentrations were calculated from a standard curve attained by the standards delivered by the manufacturer’s kit. Standard concentration values were fitted using a sigmoidal 3-parameter hill fit equation (f = a×x^b^/(c^b^ + x^b^)). Final concentrations were adapted according to the dilution factors used in the correspondent assay. For subsequent statistical analysis, log transformed data were used.

### 4.5. Statistical Analyses

Statistical analyses were conducted using R, v3.6.3 [43]. Measurements were transformed as described in [44] prior to log and z-transformation for subsequent analysis. Time-to-event data was censored after 30 months. Median follow-up data was computed with the inverse Kaplan–Meier method. Uni- and multivariate survival analyses were performed with Cox-PH or parametric survival regression models, assuming log-logistic distributed data. Differences between subgroups were calculated with linear models and Tukey’s post hoc tests. Associations between diagnosis groups and patient characteristics were evaluated using ANOVA or chi-squared tests for categorical and continuous variables, respectively. Significance level alpha was set to 5% (two-sided).

## 5. Conclusions

Our study demonstrates that profibrotic biomarkers are elevated in ATTR-CA and associated with adverse outcomes. While classical predictors such as NT-proBNP and hsTNT remain the strongest independent prognosticators, cluster-based analysis of fibrosis-associated biomarkers identifies high-risk subgroups beyond established staging systems. Prediction modeling identified eight parameters (MMP-7 TIMP-2, IGFBP-1,3,4,6, RAGE/AGE and FGF-23) as particularly relevant for cluster assignment, enabling potential clinical application with a reduced number of parameters. These results suggest that composite biomarker signatures may provide additional biological and prognostic insights, warranting validation in larger multicenter cohorts and in the context of novel anti-amyloid and antifibrotic therapies.

## Figures and Tables

**Table 1:** Patient characteristics.

	Study Population (*n* = 125)	Asympt ATTRv (*n* = 14)	ATTRv (*n* = 47)	Ctrl (*n* = 21)	ATTRwt (*n* = 43)	*p*-Value
Age (years)		43.6 ± 11.5	63.2 ± 8.3	47.8 ± 14.9	76.0 ± 7.1	0.048
Sex						
	Male	9 (64.3%)	35 (74.5%)	10 (47.6%)	40 (93.0%)	
	Female	5 (35.7%)	12 (25.5%)	11 (52.4%)	3 (7.0%)	
BMI		27.8 ± 5.4	25.9 ± 4.9	26.5 ± 5.8	25.4 ± 3.0	0.49
medication						
	Tafamidis	0 (0%)	23 (48.9%)	0 (0%)	1 (2.3%)	<0.001
	Beta blocker	2 (14.3%)	17 (36.2%)	7 (33.3%)	30 (69.8%)	
	ACE inhibitors/AT1 antagonists	3 (21.4%)	15 (31.9%)	7 (33.3%)	30 (69.8%)	
	Diuretics	2 (14.3%)	23 (48.9%)	4 (19.0%)	41 (95.3%)	
	Other antihypertensive medication (amlodipine, doxacor, nitrendipin)	2 (14.3%)	1 (2.1%)	0 (0%)	5 (11.6%)	
Comorbidities						
Coronary heart disease (CHD)		1 (7.1%)	1 (2.1%)	2 (9.5%)	7 (16.3%)	0.24
Diabetes		0 (0%)	3 (6.4%)	0 (0%)	8 (18.6%)	0.02
Thrombembolism/Stroke		0 (0%)	3 (6.4%)	0 (0%)	5 (11.6%)	0.15
Functional impairment						
	Karnofsky performance score (KPS)					0.75
	≥80	14 (100%)	35 (75.5%)	21 (100%)	42 (97.7%)	
	<80	0 (0.0%)	12 (25.5%)	0 (0%)	1 (2.3%)	
	NYHA class					0.62
	I	15 (100%)	20 (42.6%)	17 (81.0%)	5 (11.6%)	
	II	0 (0.0%)	13 (27.7%)	3 (14.3%)	12 (27.9%)	
	III/IV	0 (0.0%)	14 (29.8%)	1 (4.8%)	26 (60.5%)	
Risk Classification						
	Gillmore					0.002
	I	14 (100%)	26 (55.3%)		13 (30.2%)	
	II	0 (0.0%)	14 (29.8%)		18 (41.9%)	
	III	0 (0.0%)	9 (19.1%)		12 (27.9%)	
Medical history						
	Pacemaker implantation	0 (0.0%)	8 (17.0%)	1 (4.8%)	9(20.9%)	0.07
	Diabetes mellitus	0 (0.0%)	3 (6.4%)	0 (0%)	8 (18.6%)	0.042
	Atrial fibrillation	1 (7.1%)	14 (29.8%)	0 (0%)	26 (60.5%)	0.001
ECG findings						
	Number of bundle branch blocks	0.15 ± 0.38	0.70 ± 0.9	0.19 ± 0.51	1.2 ± 0.8	<0.001
	Sinus rhythm	14 (92.9%)	34 (72.3%)	19 (90.5%)	23 (53.5%)	
	Pacemaker rhythm	0 (0.0%)	4 (8.5%)	0 (0%)	6 (14.0%)	
	Low voltage pattern	2 (14.3%)	9 (19.1%)	0 (0%)	5 (11.6%)	
	Heart frequency (bpm)	67.5 ± 14.0	73.7 ± 15.1	47.8 ± 14.9	78.5± 13.8	0.22
	PQ interval (ms)	143.1 ± 31.2	175.9 ± 39.9	159.4 ± 24.9	212.4 ± 43.3	0.40
	QRS time (ms)	100.8 ± 16.8	110.2 ± 29.9	97.8 ± 12.5	128.7 ± 32.5	0.50
	QTc duration (ms)	404.2 ± 14.9	432.4 ± 44.4	401.6 ± 13.3	450.0 ± 31.6	0.93
Echocardiography						
	Posterior wall (mm)	9.6 ± 1.9	13.9 ± 3.8	9.6 ± 1.2	15.5 ± 3.3	0.16
	IVS (mm)	11.1 ± 1.9	16.4 ± 4.8	11.1 ± 1.0	19.4 ± 3.9	0.073
Ejection fraction (%)		58.6 ± 2.2	50.4 ± 1.5	55.4 ± 8.2	45.0 ± 11.3	0.62
	Diastolic dysfunction	2 (14.3%)	35 (74.5%)	3 (14.3%)	40 (93.0%)	<0.001
	Global longitudinal strain	−14.6 ± 14.2	−12.4 ± 1.0	n.b.	−9.8 ± 4.5	0.80
	MAPSE (mm)	1.5 ± 0.3	1.1 ± 0.4	1.5 ± 0.3	0.9 ± 0.3	0.93
	TAPSE (mm)	2.4 ± 0.4	2.0 ± 0.3	2.1 ± 0.4	1.5 ± 0.6	0.94
	Pericardial effusion	0 (0.0%)	3 (6.4%)	0 (0%)	4 (9.3%)	0.50
	PA pressure (mmHg)	26.0 ± 4.4	35.4 ± 1.8	26.9 ± 3.7	36.7 ± 10.3	0.73
Biomarkers						
	NT-proBNP(ng/L)	144.0 ± 245.6	3319.9 ± 605.1	299.2 ± 382.6	7331.2± 8593.9	0.041
	hsTNT (pg/mL)	4.8 ± 2.5	59.0 ± 15.9	7.6 ± 8.0	68.3 ± 43.4	<0.001
GFR (mL/min)		100.1 ± 15.2	77.6 ± 25.1	98.7 ± 19.0	54.0 ± 19.6	0.064

## Data Availability

All data generated or analyzed during this study are included in this published article and its Supplementary Materials. Further inquiries can be directed to the corresponding authors.

## Supplementary Materials

The following supporting information can be downloaded at https://www.mdpi.com/article/10.3390/ijms262110714/s1.

## Abbreviations

The following abbreviations are used in this manuscript:

ATTR	Transthyretin Amyloidosis
CM	Cardiomyopathy
ATTRWT	Wild-type Amyloidosis
ATTRv	Hereditary transthyretin Amyloidosis
MMP	Matrix Metalloproteinase
ENRAGE	Glycation End-Products Binding Protein
IGFBP	Insulin-Like Growth Factor Binding Protein
FGF	Fibroblast Growth Factor
TIMP	Tissue Inhibitor of Metalloproteinases
RAGE	Receptor for Advanced Glycation Endproducts
AGE	Advanced Glycation Endproducts
EDD	Enddiastolic Diameter
IVS	Intraventricular Septum
HFpEF	Heart Failure with Preserved Ejection Fraction
HFrEF	Heart Failure with Reduced Ejection Fraction
DMP	Decompensation
HTX	Heart Transplantation
CMR	Cardiac Magnetic Resonance Tomography
ECV	Extracellular Volume
NT-proBNP	N-Terminal Prohormone of Brain Natriuretic Peptide
hsTNT	High-Sensitivity Troponin T
GFR	Glomerular Filtration Rate
MAPSE	Mitral Annular Plane Systolic Excursion
TAPSE	Tricuspid Annular Plane Systolic Excursion
NYHA	New York Heart Association (classification of heart failure)
ECG	Electrocardiogram
LGE	Late Gadolinium Enhancement
LVEDD	Left Ventricular End-Diastolic Diameter
BMI	Body Mass Index
RNAi	RNA Interference
GDF-15	Growth Differentiation Factor 15
