# Supplementary material for: Profibrotic Biomarkers Correlate with Clinical Presentation and Outcome in Cardiac Transthyretin Amyloidosis
Source: Int J Mol Sci. 2025 Nov 4;26(21):10714. doi: 10.3390/ijms262110714 (PMC12609947; doi:10.3390/ijms262110714)
Supplement: Supplementary file 1 [file ijms-26-10714-s001.zip › ijms-3875920-supplementary.pptx]

## Slide 1
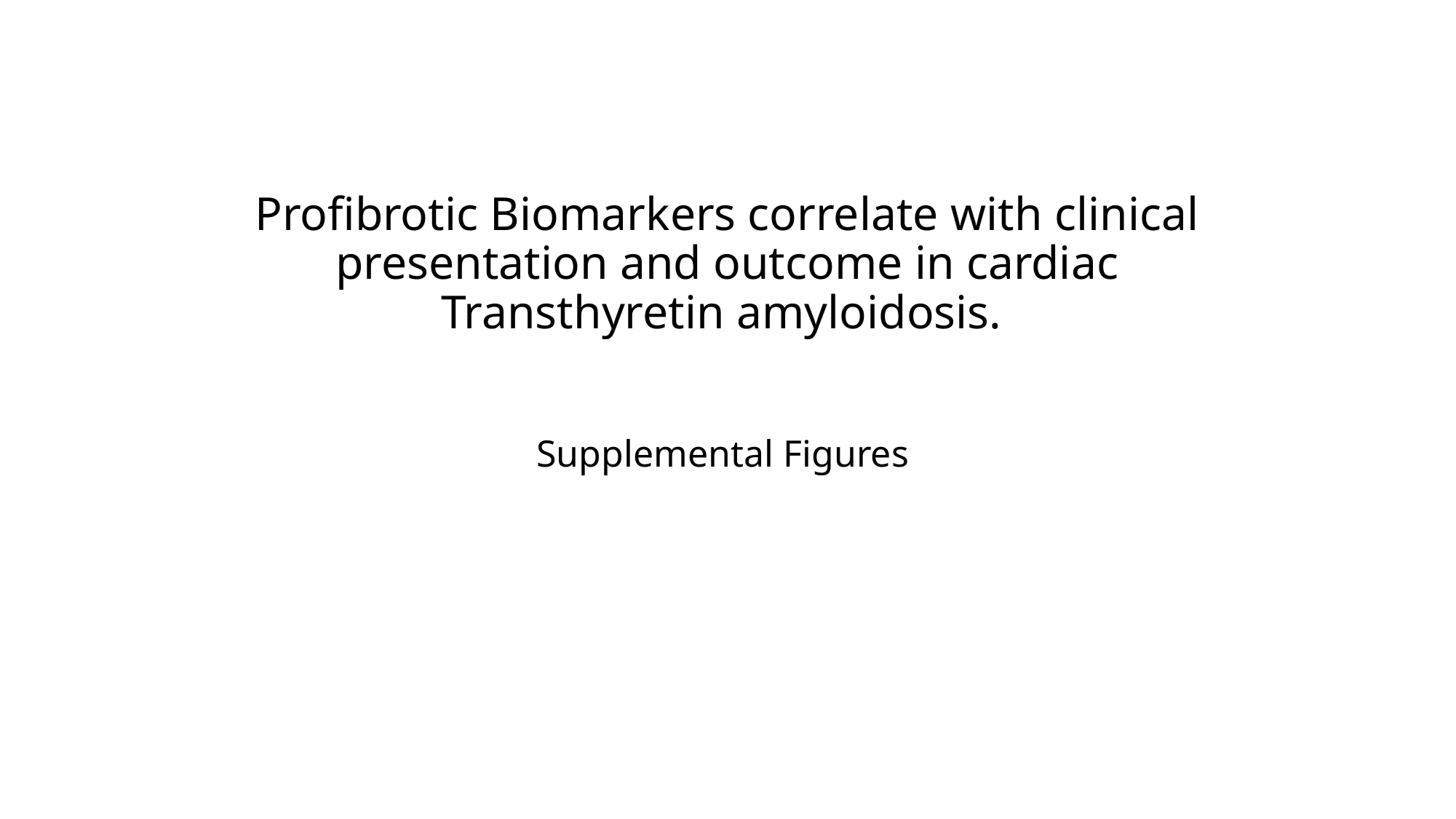

# Profibrotic Biomarkers correlate with clinical presentation and outcome in cardiac Transthyretin amyloidosis.
Supplemental Figures

## Slide 2
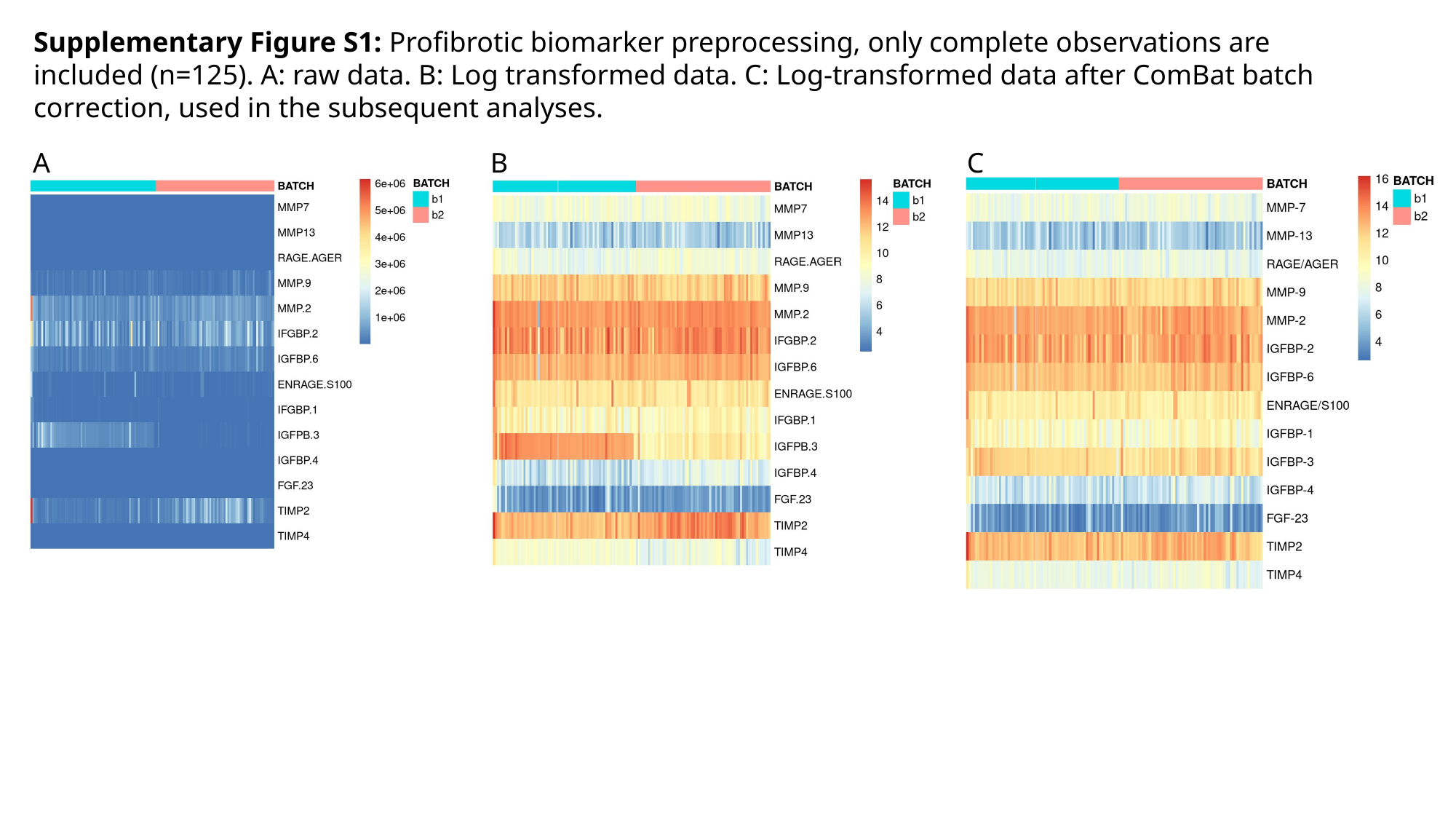

Supplementary Figure S1: Profibrotic biomarker preprocessing, only complete observations are included (n=125). A: raw data. B: Log transformed data. C: Log-transformed data after ComBat batch correction, used in the subsequent analyses.
B
C
A

## Slide 3
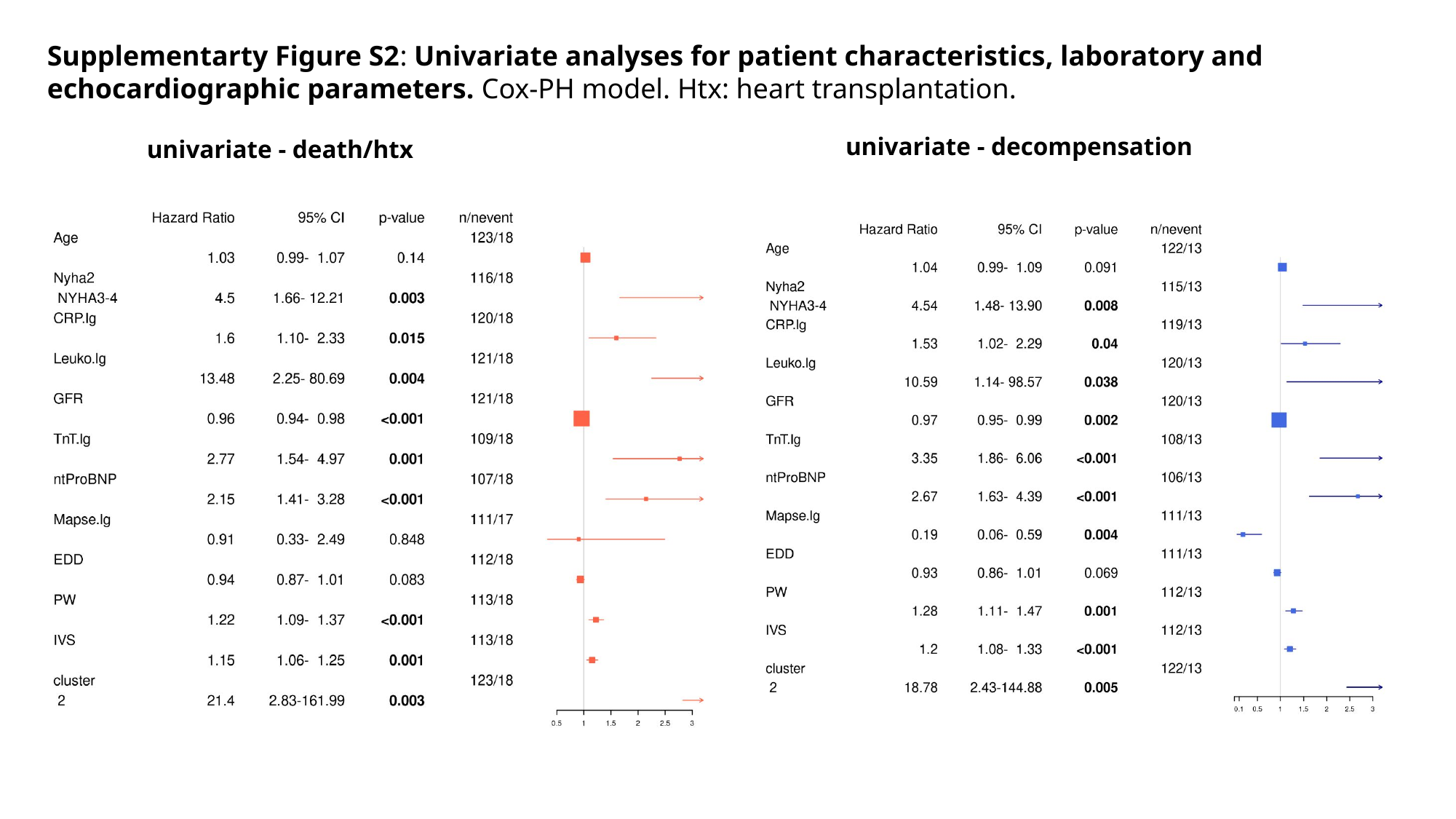

Supplementarty Figure S2: Univariate analyses for patient characteristics, laboratory and echocardiographic parameters. Cox-PH model. Htx: heart transplantation.
univariate - decompensation
univariate - death/htx

## Slide 4
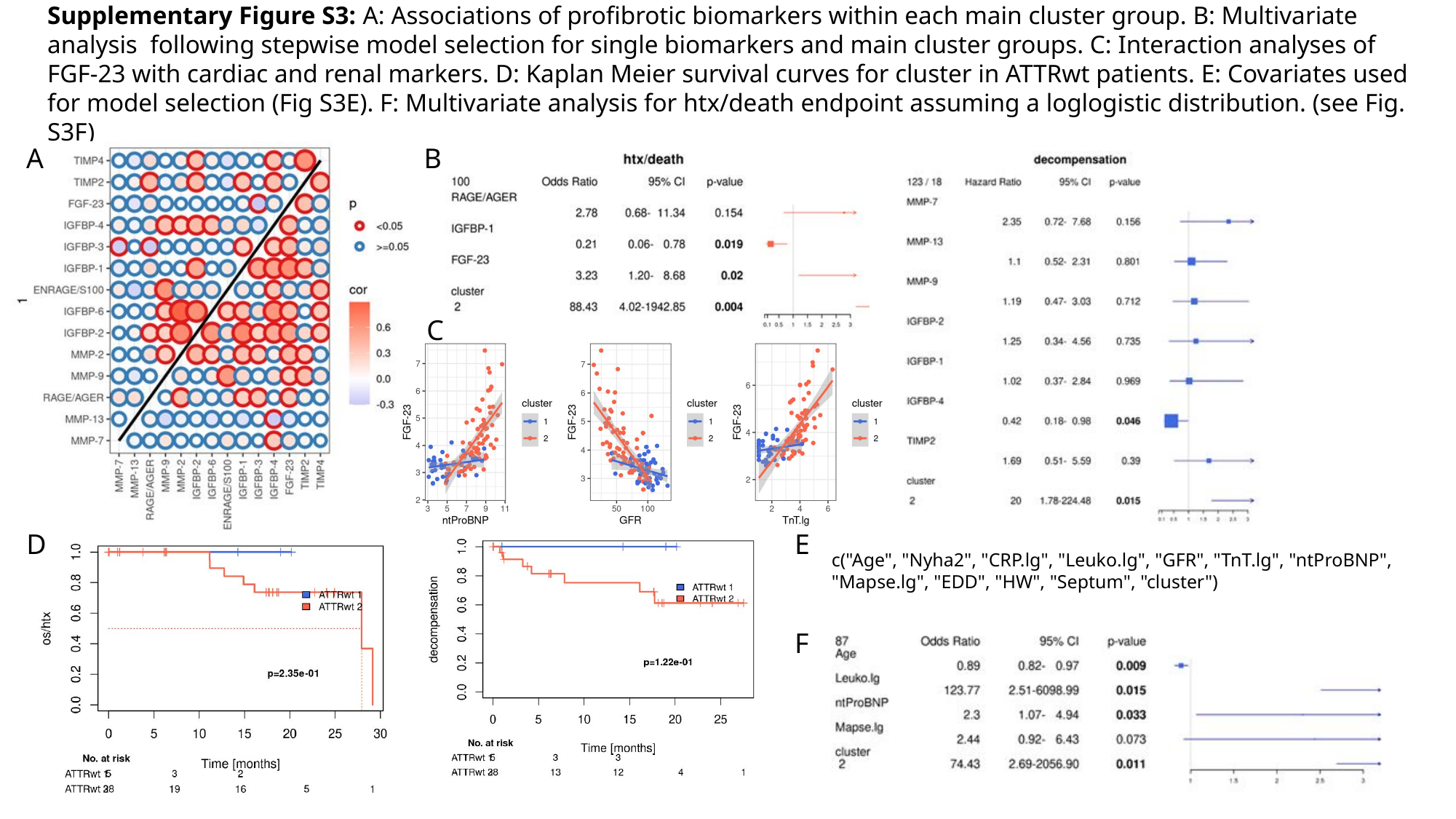

Supplementary Figure S3: A: Associations of profibrotic biomarkers within each main cluster group. B: Multivariate analysis following stepwise model selection for single biomarkers and main cluster groups. C: Interaction analyses of FGF-23 with cardiac and renal markers. D: Kaplan Meier survival curves for cluster in ATTRwt patients. E: Covariates used for model selection (Fig S3E). F: Multivariate analysis for htx/death endpoint assuming a loglogistic distribution. (see Fig. S3F)
B
A
C
D
E
c("Age", "Nyha2", "CRP.lg", "Leuko.lg", "GFR", "TnT.lg", "ntProBNP", "Mapse.lg", "EDD", "HW", "Septum", "cluster")
F

## Slide 5
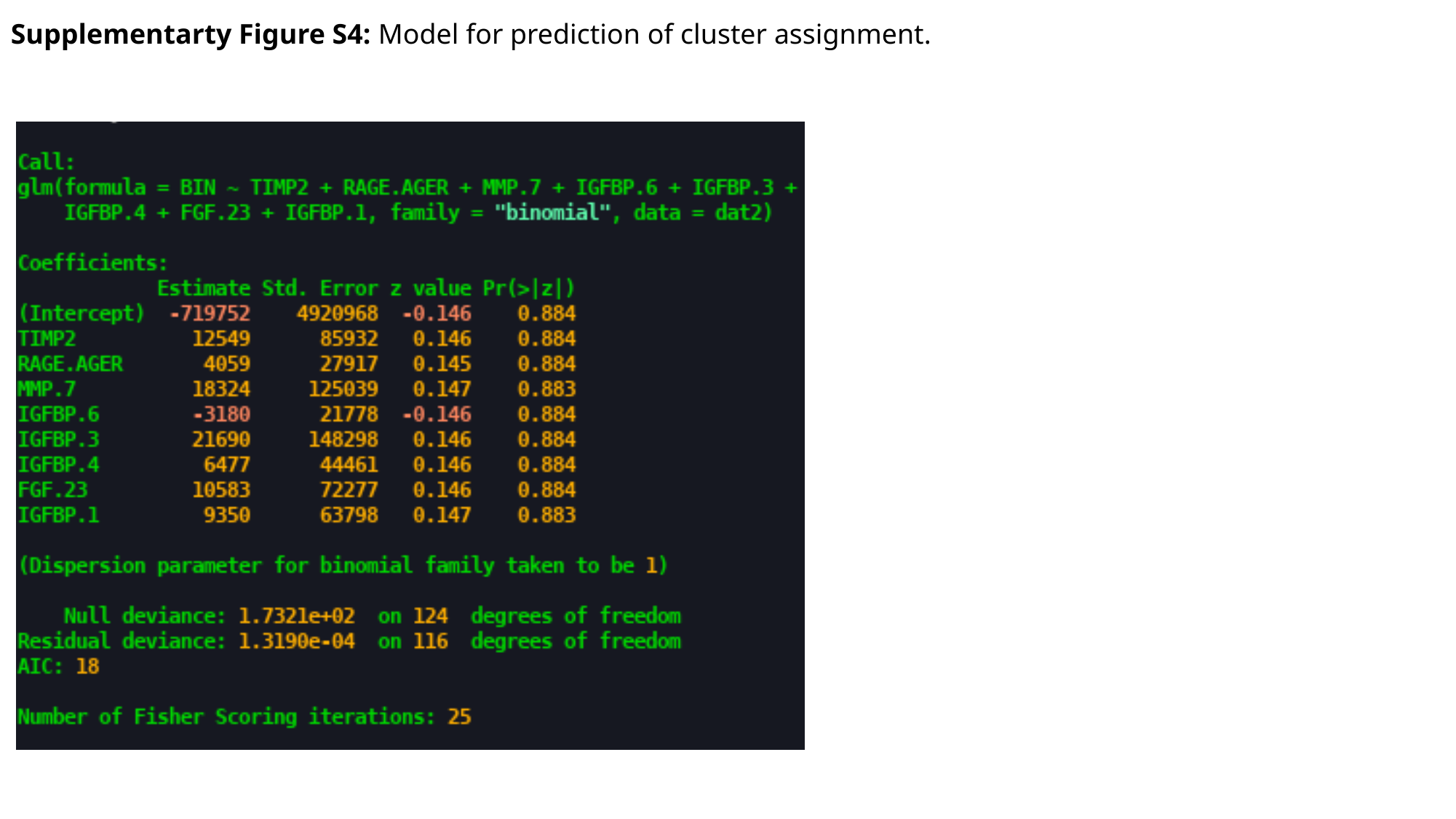

Supplementarty Figure S4: Model for prediction of cluster assignment.

## Slide 6
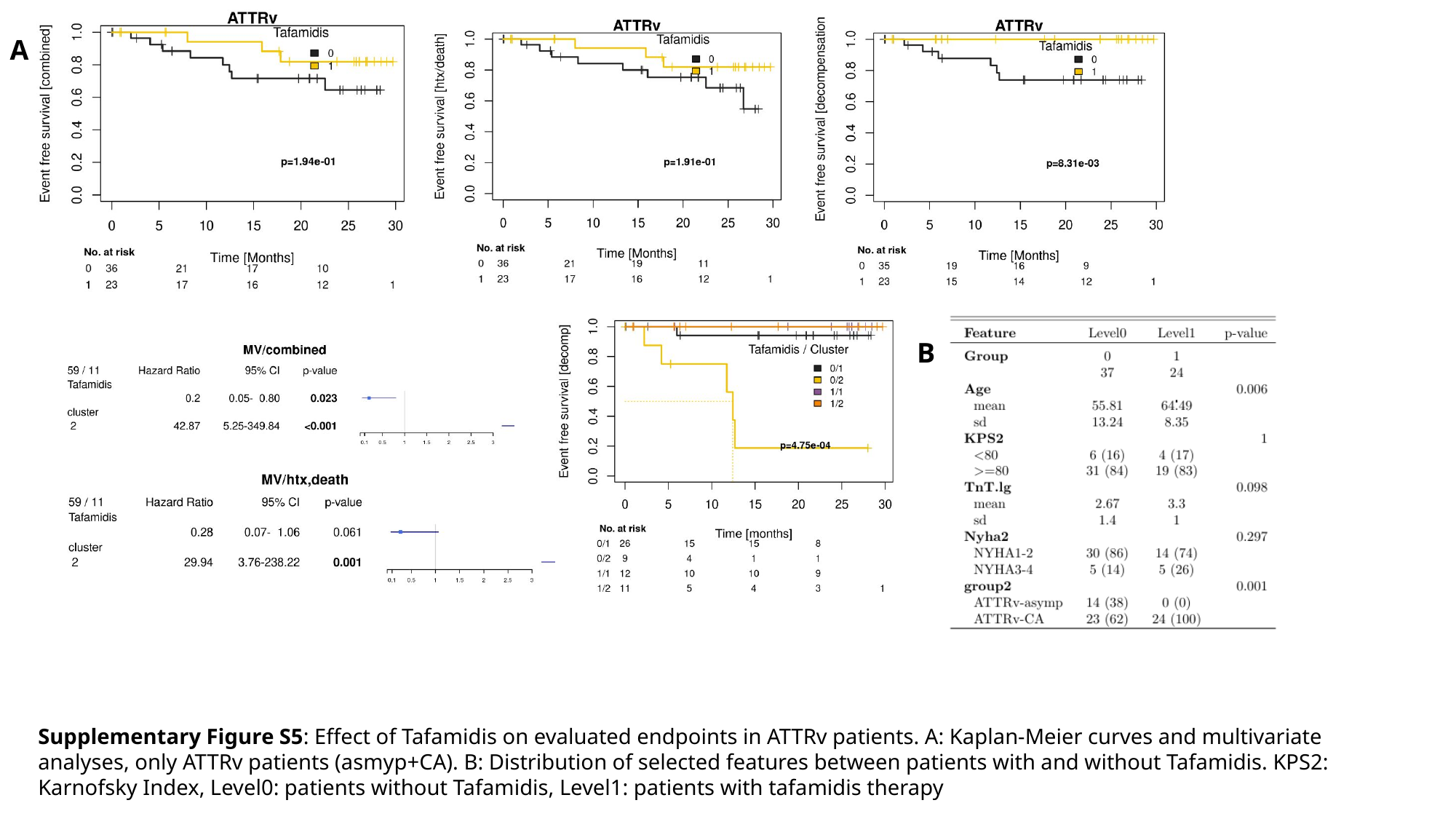

A
B
Supplementary Figure S5: Effect of Tafamidis on evaluated endpoints in ATTRv patients. A: Kaplan-Meier curves and multivariate analyses, only ATTRv patients (asmyp+CA). B: Distribution of selected features between patients with and without Tafamidis. KPS2: Karnofsky Index, Level0: patients without Tafamidis, Level1: patients with tafamidis therapy

## Slide 7
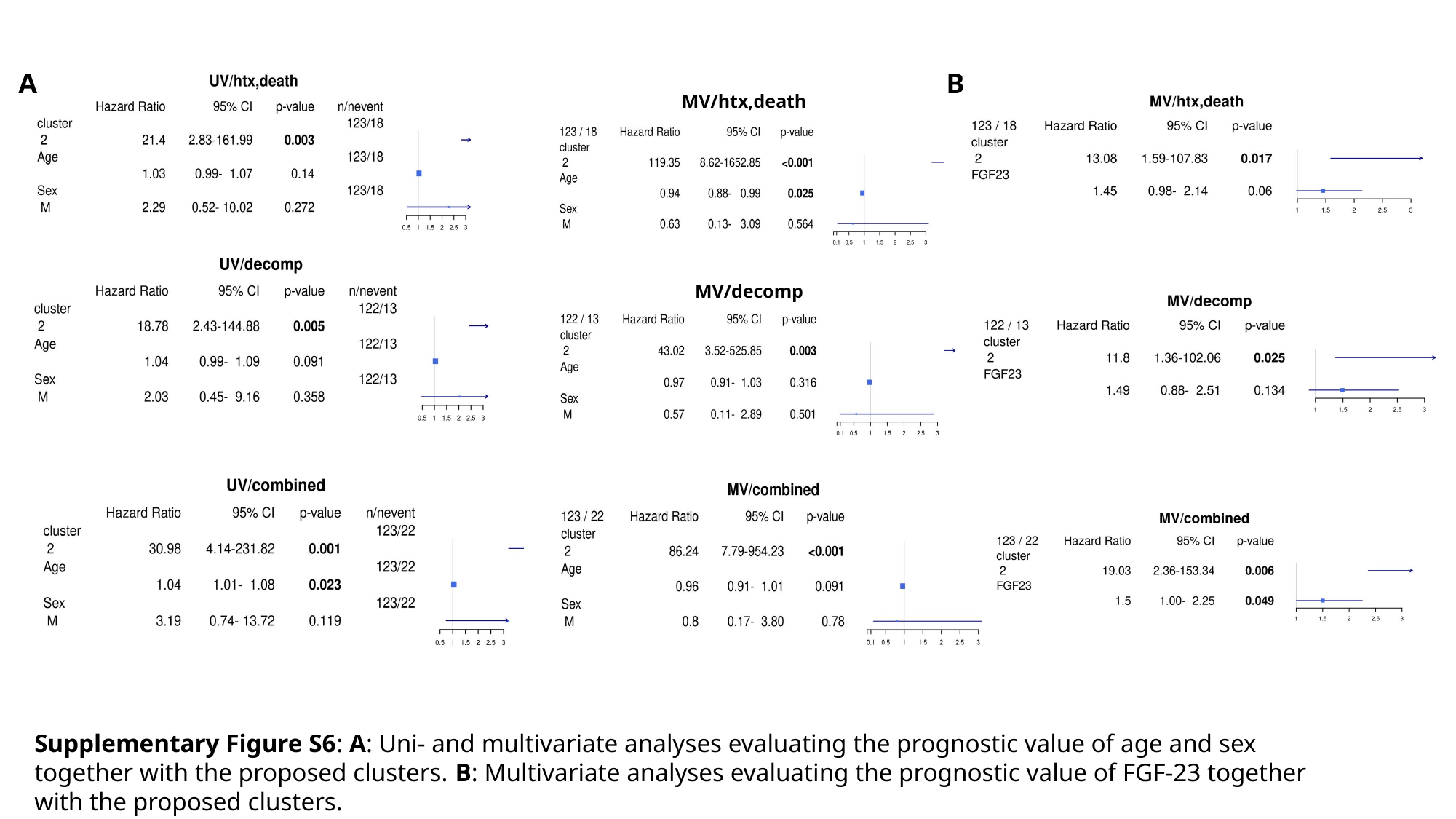

A
B
MV/htx,death
MV/decomp
Supplementary Figure S6: A: Uni- and multivariate analyses evaluating the prognostic value of age and sex together with the proposed clusters. B: Multivariate analyses evaluating the prognostic value of FGF-23 together with the proposed clusters.

## Slide 8
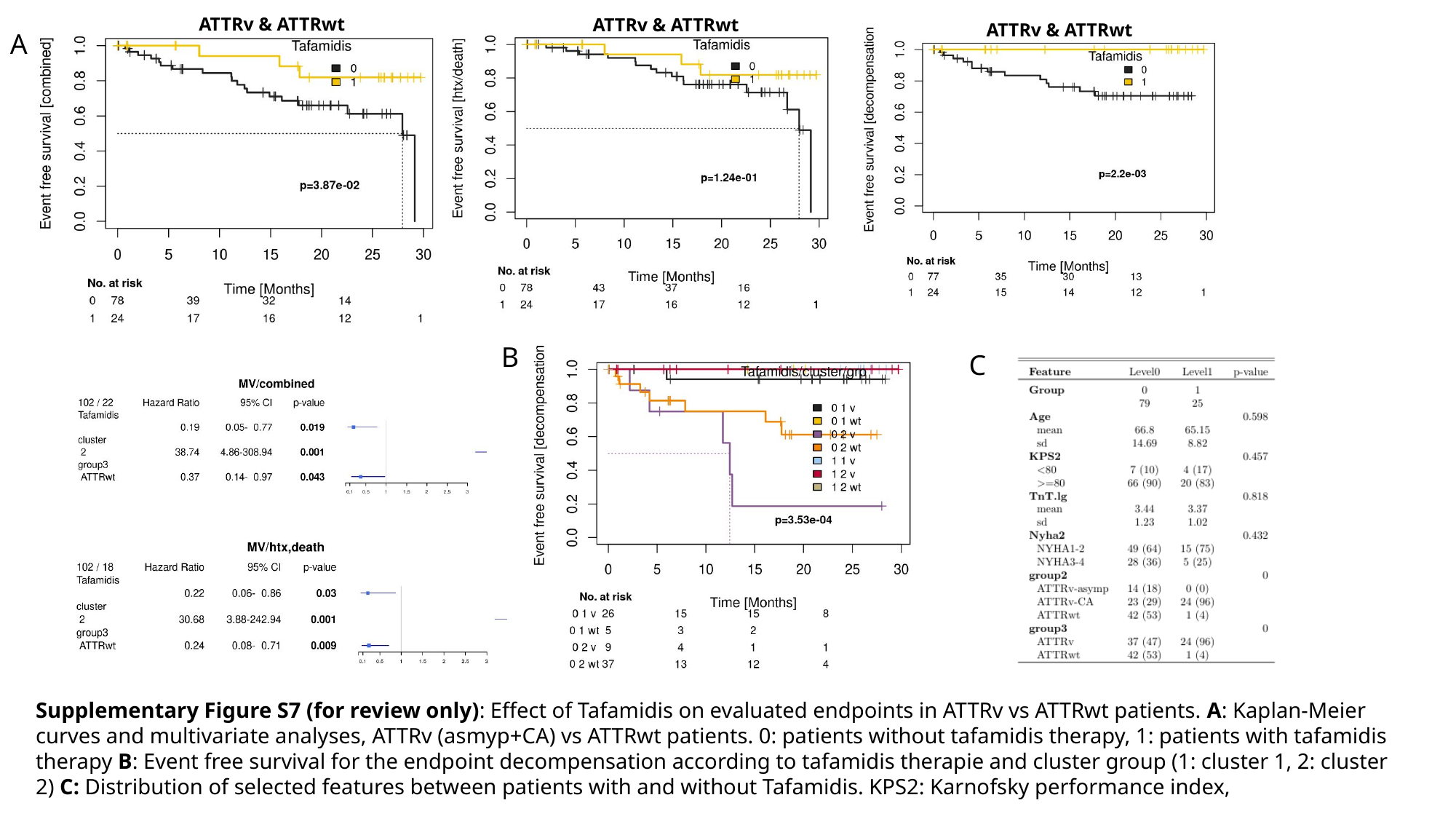

ATTRv & ATTRwt
ATTRv & ATTRwt
ATTRv & ATTRwt
A
B
C
Supplementary Figure S7 (for review only): Effect of Tafamidis on evaluated endpoints in ATTRv vs ATTRwt patients. A: Kaplan-Meier curves and multivariate analyses, ATTRv (asmyp+CA) vs ATTRwt patients. 0: patients without tafamidis therapy, 1: patients with tafamidis therapy B: Event free survival for the endpoint decompensation according to tafamidis therapie and cluster group (1: cluster 1, 2: cluster 2) C: Distribution of selected features between patients with and without Tafamidis. KPS2: Karnofsky performance index,
